# Preoperative Spinal Arterial Supply Mapping Using Non-Selective Cone Beam Computed Tomography before Complex Aortic Repair

**DOI:** 10.3390/jcm13030796

**Published:** 2024-01-30

**Authors:** Baptiste Bonnet, Hicham Kobeiter, Lorenzo Pescatori, Youssef Zaarour, Wafa Boughanmi, Mario Ghosn, Frédéric Cochennec, Nicolas Mongardon, Pascal Desgranges, Vania Tacher, Haytham Derbel

**Affiliations:** 1 Service D’imagerie Médicale Diagnostique et Interventionnelle, DMU FIxIT, Hôpitaux Universitaires Henri Mondor, Assistance Publique-Hôpitaux de Paris (AP-HP), F-94010 Creteil, France; 2 Faculté de Santé, Université Paris Est-Créteil, F-94010 Creteil, France; 3 Institut Mondor de Recherche Biomédicale-Inserm U955 Équipe 8, F-94010 Creteil, France; 4 Service de Chirurgie Vasculaire, DMU CARE, Hôpitaux Universitaires Henri Mondor, Assistance Publique-Hôpitaux de Paris (AP-HP), F-94010 Creteil, France; 5 Service D’anesthésie-Réanimation Chirurgicale, DMU CARE, Assistance Publique-Hôpitaux de Paris (AP-HP), Hôpitaux Universitaires Henri Mondor, F-94010 Creteil, France; 6 Institut Mondor de Recherche Biomédicale-Inserm U955 Équipe 3 “Pharmacologie et Technologies Pour les Maladies Cardiovasculaires (PROTECT)”, Inserm, Université Paris Est Créteil (UPEC), Ecole Nationale Vétérinaire d’Alfort (EnVA), F-94700 Maisons-Alfort, France; 7 Institut Mondor de Recherche Biomédicale-Inserm U955 Équipe 18, F-94010 Creteil, France

**Keywords:** computed tomography angiography, cone beam computed tomography, Adamkiewicz artery, aorta, endovascular procedures

## Abstract

Pre-op spinal arterial mapping is crucial for complex aortic repair. This study explores the utility of non-selective cone beam computed tomography (CBCT) for pre-operative spinal arterial mapping to identify the Adamkiewicz artery (AKA) in patients undergoing open or endovascular repair of the descending thoracic or thoracoabdominal aorta at risk of spinal cord ischemia. Pre-operative non-selective dual-phase CBCT after intra-aortic contrast injection was performed in the aortic segment to be treated. The origin of detected AKA was assessed based on image fusion between CBCT and pre-interventional computed tomography angiography. Then, the CBCT findings were compared with the incidence of postoperative spinal cord ischemia (SCI). Among 21 included patients (median age: 68 years, 20 men), AKA was detected in 67% within the explored field of view, predominantly from T7 to L1 intercostal and lumbar arteries. SCI occurred in 14%, but none when AKA was not detected (*p* < 0.01). Non-selective CBCT for AKA mapping is deemed safe and feasible, with potential predictive value for post-surgical spinal cord ischemia risk. The study concludes that non-selective aortic CBCT is a safe and feasible method for spinal arterial mapping, providing promising insights into predicting post-surgical SCI risk.

## 1. Introduction

Spinal cord ischemia (SCI) is a rare but severe complication that can occur during suprarenal aortic repair, whether it is performed through open (OR) or endovascular (ER) approaches. This complication is often referred to as the “Russian roulette complication” among vascular surgeons due to its unpredictability [1,2,3]. While the occurrence of SCI is unpredictable, an acute loss of blood flow to the anterior spinal artery (ASA) has been identified as a significant risk factor for this condition [4]. The most critical feeding artery of the thoracolumbar spinal cord is the Adamkiewicz artery (AKA), which originates on the left side in about 70% of cases at the level of the 9th–12th intercostal artery [5]. 

Due to the potential risks involved, many aortic centers routinely perform pre-treatment imaging of the spinal cord’s vascularization to plan the best surgical strategy and supportive care during interventions. This is particularly important if the AKA’s feeders are excluded from the aortic circulation during the treatment [6,7]. Various strategies exist to minimize the risk of SCI and consequent paraplegia, such as sparing or reimplantation of the artery of interest, pre-operative drainage of cerebrospinal fluid, motor evoked potential monitoring during surgery, and controlled augmentation of blood pressure during and after the procedure [8,9]. 

Several imaging techniques have been proposed to detect the AKA and its feeders, including selective digital subtraction angiography (DSA), magnetic resonance-angiography (MRA), and computed tomography-angiography (CTA) [9,10,11,12,13,14,15]. However, these modalities have some limitations that may affect their effectiveness. For instance, DSA can be hard to perform in patients with aortic aneurysm or dissection disease and can have a non-negligible complication rate, while MRA poses certain limitations due to claustrophobia or intra-body metallic/electronic devices, and CTA may have some degree of uncertainty in the differential diagnosis between the AKA and the great anterior radiculomedullary vein (GARV) [9,10,11,12,13,14,15]. 

Some studies have described an intra-arterial non-selective computed tomography angiography (CTA) technique with a high rate of Adamkiewicz artery (AKA) detection [16,17]. However, this approach poses some feasibility issues, such as requiring hybrid interventional equipment or transferring patients from the angiography suite to the CT room, which may lead to a loss of asepsis or catheter migration. More recently, the usefulness of contrast-enhanced cone beam computed tomography (CBCT) has been demonstrated in characterizing vascular patterns of spinal vascular malformations or spinal dural arteriovenous fistulas [18]. However, no previous studies have examined the potential of CBCT in depicting the path of the anterior spinal artery (ASA) and its feeder for aortic surgeries. 

Therefore, the purpose of this study is to evaluate the feasibility, efficacy, and safety of CBCT in detecting the AKA for patients scheduled for open or endovascular repair of the descending thoracic or thoracoabdominal aorta.

## 2. Materials and Methods

### 2.1. Study Design and Population

The local institutional review board approved this retrospective, single-center study (CRM-2312-382). According to the retrospective design and the non-interventional nature of the study, written informed consent was waived. Between January 2015 and July 2019, we included all patients planned for a thoracic descending or thoracoabdominal aortic repair being at high risk for spinal ischemia, e.g., with a minimum of 20 cm of aorta meant to be treated or patients who already had an endovascular aortic repair and who needed a stent-graft extension to the supra-renal or infra-renal aorta. Patients underwent these procedures when AKA mapping could not be established on previous CTA.

Therapeutic decisions were discussed in a multidisciplinary board meeting between radiologists, vascular surgeons, and anesthesiologists. According to the aortic repair plan, AKA was pre-operatively investigated in the aortic segment to be treated. As the procedures performed were the standard of care in our center, patients were informed initially of the procedure’s objectives, benefits, and risks, and they provided oral consent. 

### 2.2. Procedure and CBCT Data Acquisition

All interventions were performed in two angiographic suites with a flat panel detector C-arm angiographic system (Allura Clarity^®^/Azurion Clarity^®^; Phillips Healthcare, Best, The Netherlands).

Patients were placed in a supine position on the angiographic table. The right femoral or left brachial artery was punctured by the Seldinger technique under local anesthesia (lidocaine 1%), and a 4 French introducer sheath was then inserted. The aorta was catheterized with a 4 French Pigtail-shaped catheter (Cordis Corporation, Baar, Switzerland), positioned at the proximal end of the aortic segment meant to be analyzed. Then, a dual phase-CBCT acquisition was launched with the C-arm at a head position. The dual phase-CBCT protocol consisted of acquiring two consecutive rotations after a single non-selective injection of contrast agent through the Pigtail catheter. The first CBCT acquisition (launched 3 s after the beginning of the contrast agent’s injection) was meant to show the spinal arterial network, while the second CBCT acquisition (15 s after the beginning of the contrast agent’s injection) was supposed to depict the AKA better and to distinguish it from the GARV.

CBCT acquisition parameters were as follows: a single CBCT scan involved a 5-s rotation acquiring 312 frames (60 frames/s) and covering a 240° clockwise arc in a propeller axis rotation of the C-arm with the X-ray tube traveling around the patient, with a tube voltage of 120 kVp and a tube current of 200–300 mAs. The flat panel detector displayed a field of view (FOV) of 250 × 250 × 193 mm with a matrix size of 384 × 384 × 297 pixels. Acquired CBCT data had an isotropic resolution of 0.65 mm. 

The protocol of injection of contrast agent was standardized; volumes and injection rate were as follows: 40 mL of nonionic iodinated contrast medium, [iodixanol 320 mg/mL (Visipaque^®^; GE Healthcare AS, Oslo, Norway)] injected at a rate of 5 mL/s through a mechanical contrast injector (Medrad^®^, Bayer AG, Leverkusen, Germany). Each CBCT acquisition was obtained during a single breath-hold with free breathing between the two phases to avoid motion artifacts and ensure optimal image quality and patient comfort. The acquisition was repeated if the CBCT FOV was too narrow to cover all the aortic segments of interest.

CBCT images were automatically transferred to a dedicated 3D workstation (XtraVision Release 8^®^; Philips Healthcare, Best, The Netherlands). A first image evaluation was performed right after the acquisition by an experienced interventional radiologist to assess the quality and to repeat the acquisition if needed. In case of unsatisfying aortic enhancement on the first acquisition, the CBCT was repeated, increasing the injection rate to 7 mL/s.

### 2.3. Post-Processing and Image Analysis

**Figure 1:**
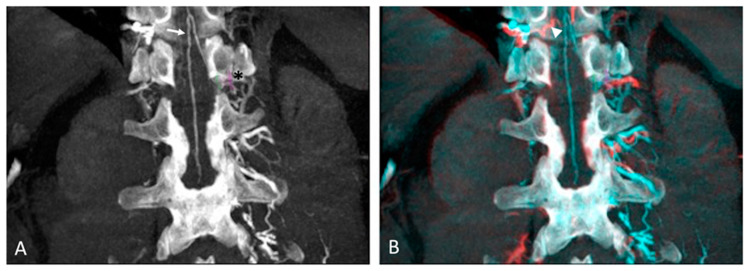
Anterior spinal and Adamkiewicz arteries detection on intra-aortic CBCT coronal images with maximum intensity projection: (**A**) Arterial phase of CBCT showing the anterior spinal artery with its characteristic “hairpin” curved vessel (arrow), supplied by 12th left intercostal artery (Asterix). Note the purple circle placed as a landmark on the intercostal foramina. (**B**) Registered arterial and delayed phases of CBCT with identification of great anterior.

Images from the first arterial CBCT phase were analyzed to identify the AKA after multiplanar (MPR) and maximum intensity projection (MIP) reconstructions. It was identified as a characteristic “hairpin” curved vessel originating from intercostal or lumbar arteries and landing on the anterior midsagittal surface of the spine (Figure 1). Images from the second arterial CBCT phase were used to assess the absence of further enhancement of other ASA feeders differentiating the AKA from the GARV. Images were reviewed and analyzed in consensus by two experienced radiologists (HD and HK) to detect the AKA and its collateral supply.

**Figure 2:**
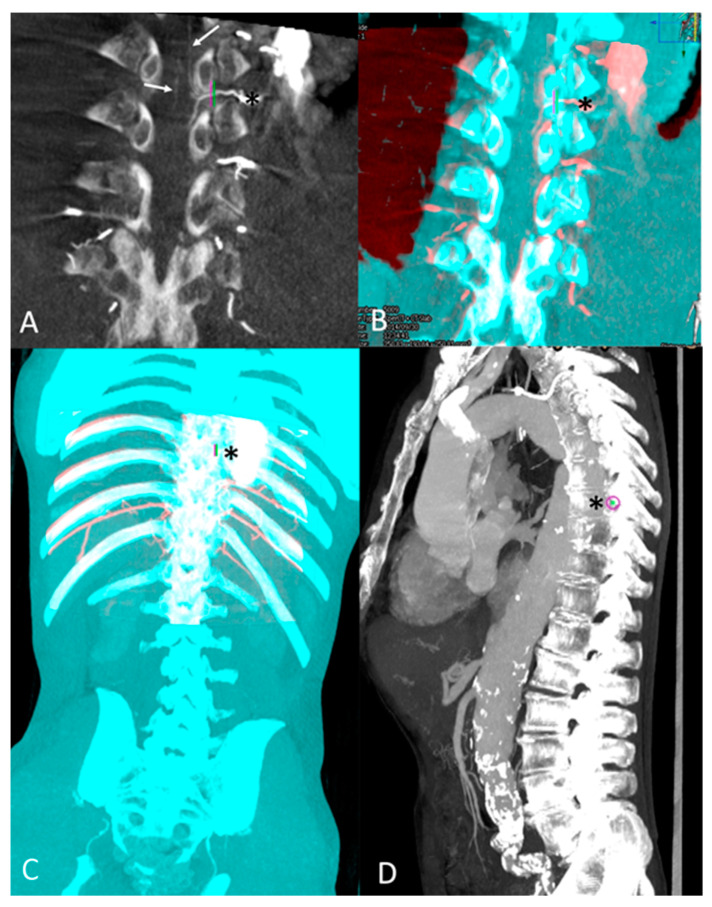
Anterior spinal detection on intra-aortic CBCT and feeder intercostal artery level tagging after 3D-3D registration with pre-operative CTA: (**A**) Coronal view of arterial phase CBCT with MIP reconstruction: detection of ASA (arrow). Its intercostal feeder (Asterix *) was marked by a landmark (green and purple). (**B**) 3D-3D semi-automatic registration with pre-procedural CTA images based on osseous structures (CBCT volume red colored; CTA volume blue colored). Its intercostal feeder (Asterix *) was marked by a landmark (green and purple). (**C**) Tagging of the ASA feeder, the 8th left intercostal artery (Asterix *), on an overview of overlaid CTA and CBCT volumes. (**D**) Global sagittal view of the entire spinal column with MIP reconstruction showing the level of 8th intercostal foramina with purple landmark (Asterix).

A 3D-3D semi-automatic registration was performed between CBCT and pre-interventional CTA images, preloaded on the posttreatment workstation, using commercially available software (XperCT^®^; Phillips Healthcare, Best, The Netherlands), as described previously [19,20]. The registration to merge CBCT and CTA images was based on vertebral bodies and aortic calcifications as landmarks. Then, the intervertebral foramen corresponding to the intercostal or lumbar feeder of the AKA was marked with a colored ring using XperGuide^®^ software (Phillips Healthcare, Best, The Netherlands). The level of the main AKA’s feeder was consequently determined by referring to the entire spine analysis (Figure 2).

### 2.4. Success Assessment

Feasibility was defined by the technical success of the procedure, which included aorta catheterization, angiography of the aortic segment to be analyzed, and the visualization of the relevant intercostal and lumbar arteries. 

Efficacy was defined as the ability of CBCT to detect the AKA and its feeder within the selected FOV.

Safety was defined based on procedural and post-procedural complications (i.e., allergic reaction to the contrast agent, puncture site hematoma or pseudoaneurysm, aortic aneurysm rupture, and iatrogenic SCI). 

Total volume injection of contrast agent, X-ray exposure (expressed as a dose-area product (DAP)), and fluoroscopy time were also collected for each patient.

Patients were followed up, and aortic surgery characteristics were recorded, including surgery technique and per-operative measures to prevent SCI. Per- and postoperative complications were also assessed.

### 2.5. Statistical Analysis

The normal distribution of quantitative variables was verified using the Kolmogorov–Smirnov test. Categorical variables were expressed as frequency, and continuous variables not normally distributed were expressed as a median and interquartile range [Q1–Q3]. Fisher exact test was used to assess the correlation between categorical variables. *p* values ≤ 0.05 were considered statistically significant. All statistical analysis was performed using SPSS Version 25.0 (SPSS, Inc., Chicago, IL, USA).

## 3. Results

### 3.1. Patient Description

Twenty-one consecutive patients (20 males, 1 female) aged between 32 and 89 years [median 68 (61–74)] were enrolled in the study. Patients were affected by descending thoracic aortic aneurysm in 1 case (5%), thoraco-abdominal aortic aneurysm in 10 cases (47%), and aortic dissection (AD) with aneurysmal evolution in 10 cases (47%): among them, six had type B dissection and four had type A (according to the Stanford classification) who already underwent an open surgery of the ascending thoracic aorta but still presented a remnant enlarging dissection of the descending aorta. OS was planned in 9 cases (43%) and ER in 12 cases (57%). Among patients planned to have an ER, 10 patients (48% out of the whole population and 83% out of the ER subgroup) underwent an endograft extension, either for an aneurysmal evolution over an aortic dissection (in 6 patients) or for an aneurysmal evolution of the abdominal aorta over a grafted thoracic aneurysm (in 4 patients).

All the patients underwent arterial spinal mapping between 2 and 11 days before the aortic repair. The population characteristics are detailed in Table 1

### 3.2. Procedural Success and Complications

The procedure was feasible in 100% of cases. The puncture site was the right common femoral artery in 15 patients (71%), the left common femoral artery in 2 patients (10%), and the left brachial artery in 4 patients (19%).

All the CBCTs’ images were acquired to investigate the aortic segment to be treated according to the surgical planification (Table 2). In 13 cases, the CBCT FOV did not cover all the aortic segments of interest, requiring a complementary second CBCT. CBCT acquisition was repeated in six cases due to the unsatisfying quality of CBCT images by respiratory motion artifacts or due to insufficient aortic enhancement.

**Figure 3:**
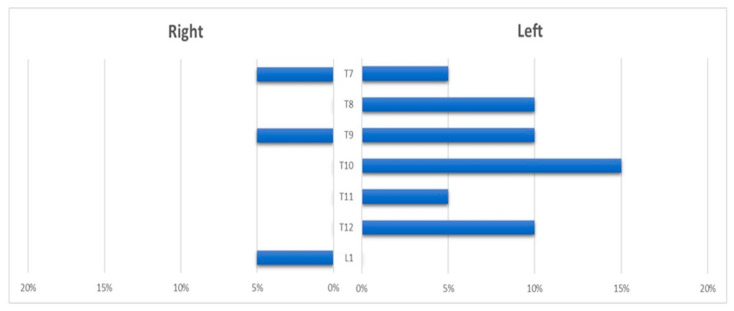
Histogram showing the distribution of the anterior spinal artery feeder origin level.

The ASA and its feeders were visualized in 14 cases (67%). The AKA had a left-sided origin in 11 patients (79%), and a right-sided origin in 3 (21%); it was fed by the intercostal arteries between T5 and T11 in 10 cases (72%) and by the lumbar arteries (T12-L1) in 3 cases (21%); multiple anastomotic feeders were found in 1 case (7%) (Figure 3). 

### 3.3. X-ray Exposure and Contrast Medium Parameters

The median DAP was 70.52 Gy.cm² [IQR: 28.53–117.39] and the median fluoroscopy time was 4 min [IQR: 1.7–7.7]; mean contrast volume injection was 87 ± 36 mL [35–140].

### 3.4. Safety and Patient Follow-Up 

One pseudo-aneurysm at the puncture site of the brachial artery, needing surgical repair, was recorded. No other complications were observed. 

Patients underwent specific spinal protection measures during the aortic surgery (Table 3), i.e., cerebrospinal fluid drainage in 14 cases (100% of the group where the AKA was found), AKA reimplantation in 4 cases if OS was performed and the graft was considered technically feasible, and MEP monitoring in 6 cases. In addition, maintaining high blood pressure levels was a part of the anesthetic protocol of every aortic repair surgery in our center. 

Irreversible SCI occurred in three cases (14%) and transient SCI occurred in one case (5%) with spontaneous resolution after 15 days. In two cases, SCI occurred immediately after the aortic intervention, and it was diagnosed directly in the recovery room or during the post-operative hospitalization. In the other two cases, SCI was triggered by an extended time of low blood pressure due to septic shock. Nevertheless, in one case, SCI symptoms recovered fully after treating the shock, and the patients completely recovered at hospitalization check-out.

No patient in whom AKA was not identified in the aortic segment of interest presented postoperative SCI. Post-operative SCI was significantly correlated to AKA detection on CBCT (*p* < 0.001) and to internal iliac arteries patency (*p* = 0.025). No significant correlation was found between SCI and general risk factors (*p* = 0.54) or type of surgery (*p* = 0.32).

## 4. Discussion

The present preliminary study highlights the feasibility of 100% with a detection rate of 67% for ASA within the investigated aortic segment using nonselective CBCT before aortic repair. 

Spinal arterial mapping with nonselective enhanced CBCT in the potential aortic segment to treat may be sufficient to ensure the absence of complications related to AA exclusion. In the seven cases in which the ASA was not visualized, no further investigations were planned as we assumed that any origin of the AKA over a lumbar artery other than those meant to be involved in the intervention would not have directly caused SCI by abruptly interrupting the vascular supply to the ASA. Even though a definitive statement cannot be proposed, considering the absence of any gold standard for comparison, we found that no SCI occurred in those cases where AKA was not visualized. Given these findings, we can hypothesize two scenarios: (i) the AKA originated at a lower or upper level through some collateral vessels that were not meant to be concerned by the intervention; (ii) the diameter of the AKA was too small to allow a contrast opacification, in which case, it was probably too small to be the main feeder vessel to the ASA as well. Moreover, the relatively high rate of patients treated for type B dissection can explain the low rate of AKA visualization, as the intimal flap can be responsible for hemodynamic modifications causing redistribution of the ASA’s flow through a collateral network [21].

Interestingly, our study found a higher post-treatment SCI rate than the literature [1,2,3,4]. This finding could be explained by the heterogeneity of the underlying aortic diseases and the complexity of the aortic repair procedures in our series. Eighteen patients (86%) had a severe underlying pathology (i.e., extensive dissection or large thoracoabdominal aneurysms) and, among them, ten patients (48%) underwent two or multiple treatments of the thoracic aorta, with a consequent increased SCI risk [4]. 

In our study, no major complication occurred during the CBCT examination, especially aortic aneurysm rupture, SCI, or stroke, known as the main severe complications of selective conventional spinal DSA [8]. Repeated CBCTs were often needed to achieve sufficient aortic length coverage with limited FOV. However, procedures were performed with less contrast injection and less X-ray exposure, which is particularly interesting for the target population, typically burdened with severe renovascular impairment [22] and supposed to undergo several radiological examinations during the follow-up [23]. The mean volume of injected contrast media in our series was 87 ± 36 mL, which is significantly less than DSA and intra-aortic CT as reported in previous studies [10,17].

The smaller detection rate of ASA in our study compared to other studies based on selective catheterization of all intercostal and lumbar arteries [10] or intra-aortic CTA [16,17] can be explained by several reasons. First, we did not explore the whole aorta but solely the relevant aortic segment to treat, neglecting ASA arising from the intercostal or lumbar artery out of the exploration field. Secondly, the relatively high ratio of patients with dissection as aortic underlying pathology could have induced hemodynamic modifications, as well as re-injection of the ASA by the collateral network, in case of occlusion of its main feeder.

This study highlights the significant variability of the AKA origin that can burden vascular surgeons during aortic repair. These variations are well-studied for arteries emerging from the thoracoabdominal aorta, implying rigorous planning of aneurysm treatments [24,25]. Given its variability of emergence, the pre-operative mapping of the AKA is all the more crucial as the consequences of its obstruction can be dramatic.

Our study has some limitations. First, the retrospective design and the low sample size preclude the possibility of stating any definitive conclusion about the predictive value of the technique. Another limitation is the absence of a control group to compare X-ray exposure and contrast media injection with the conventional selective DSA technique. Finally, the absence of a standardized consensus about the etiology of SCI and the peri-procedural measures to prevent it impedes any unequivocal statement about the value of AKA detection and a non-biased correlation with our study’s relatively high rate of post-operative SCI incidence. 

## 5. Conclusions

This study demonstrated the feasibility and efficacy of pre-operative arterial spinal mapping using nonselective CBCT before descending or thoraco-abdominal aortic repair. This technique limits contrast media injection, X-ray exposure, and complications compared to selective DSA of the intercostal and lumbar arteries, and it maintains sufficiency to confirm the absence of the AKA in the aortic segment meant to be treated. Further studies are needed to validate and confirm our preliminary results.

## Figures and Tables

**Table 1:** Patients’ characteristics, aortic repair indication, and surgery technique.

Patient	Age (Years)	Sex	Type of Aneurysm	Type of Intervention	IIAs Patency		SCI Risk Factors	
					Right	Left	HT	Diabetes
1	65	M	pararenal AAA	f-EVAR	X	X	X	X
2	74	F	TAAA	f-EVAR	X	X		X
3	89	M	suprarenal AAA	f-EVAR	X	X	X	
4	76	M	TAAA (Type B AD)	OR	X	X	X	
5	61	M	TAAA	f-EVAR	X	X	X	
6	35	M	TAA (type A AD)	f-EVAR	X	X	X	
7	70	M	TAA	TEVAR	X	X		X
8	67	M	TAAA (Type B AD)	f-EVAR			X	
9	63	M	TAAA	OR	X	X	X	
10	57	M	TAA (Type B AD)	TEVAR	X	X		X
11	68	M	TAAA	OR			X	
12	55	M	TAAA	OR	X	X	X	
13	63	M	TAAA (Type B AD)	OR	X	X	X	
14	37	M	TAAA (type A AD)	OR	X	X	X	
15	74	M	TAAA	OR	X		X	
16	75	M	TAAA	f-EVAR	X	X		
17	70	M	suprarenal AAA	f-EVAR	X	X	X	
18	32	M	TAA (Type B AD)	OR	X	X	X	
19	80	M	TAA (Type B AD)	OR	X	X	X	
20	69	M	TAAA (type A AD)	OR			X	
21	73	M	TAAA (type A AD)	f-EVAR	X	X	X	X

IIAs: internal iliac arteries; HT: hypertension; AAA: abdominal aortic aneurysm; TAA: descending thoracic aortic aneurysm; TAAA: thoraco-abdominal aortic aneurysm; AD: aortic dissection; OR: surgical open repair; TEVAR: thoracic endovascular aortic repair; f-EVAR: fenestrated endovascular aortic repair; SCI: Spinal cord ischemia.

**Table 2:** Cone beam computed tomography imaging features and spinal arterial mapping results.

Patient	Number of CBCTs	Reason for Multiple CBCTs	Contrast Rate (mL/s)	Contrast Quantity (mL)	Fluoroscopy Time (s)	Dap (Gy.cm²)	AKA Detection	AKA’s Origin
1	2	Length	5	80	5′58′′	79.47	Yes	12th ICA—L
2	2	Length	5	80	9′21′′	29.99	Yes	10th ICA—L
3	2	Length	5	80	3′28′′	22.5	No	
4	3	Length	5	120	11′56′′	120.78	Yes	9–10th ICA—L&R
5	2	Length	5	80	0′47′′	27.082	Yes	1st LA—R
6	2	Length	5	65	1′27′′	89.42	Yes	8th ICA—L
7	3	Length (x2)—quality (x1)	5	120	6′14′′	144.153	No	
8	2	Length	5	70	1′56′′	34.129	Yes	7th ICA—L
9	1		5	35	3′40′′	25.44	Yes	7th ICA—R
10	3	Quality	5, 5, 7	120	9′09′′	150.522	No	
11	2	Length	5	70	3′45′′	80.665	Yes	8th ICA—L
12	3	Length (x2)—quality (x1)	5	105	4′10′′	51.029	No	
13	3	Quality	5, 5, 6	112	4′12′′	65.125	Yes	9th ICA—L
14	4	Length (x2)—quality (x2)	5, 6	170	5′14′′	127.506	Yes	11th ICA—L
15	3	Length	5	115	4′40′′	70.52	Yes	5th ICA—L
16	1		5	40	0′52′′	21.309	no	
17	1		5	35	1′06′′	26.344	No	
18	3	Quality	5, 5, 7	120	2′20′′	58.289	No	
19	3	Length	5	110	36′31′′	83.425	Yes	9th ICA—L
20	1		5	40	20′′	114	Yes	12th ICA—L
21	1		5	40	1180′′	134	Yes	10th ICA—L

CBCT: Cone beam computed tomography; AKA: Adamkiewicz artery; DAP: dose area product; ICA: intercostal artery; LA: lumbar artery; L: left; R: right.

**Table 3:** Perioperative prophylactic measures from spinal cord ischemia.

Patient	Prophylactic Measures			Post-Operative SCI
	CSF Drainage	AKA Surgical Re-Implantation	MEP Monitoring	
#1				
#2	X			
#3	X			
#4	X	X		
#5			X	transient
#6	X		X	
#7			X	
#8	X			irreversible
#9	X			
#10				
#11		X		
#12	X			
#13				
#14	X	X		
#15	X			irreversible
#16				
#17	X		X	
#18	X			
#19	X	X	X	
#20	X		X	Irreversible
#21	X			

CSF: cerebrospinal fluid; MEP: motor evoked potentials; SCI: spinal cord ischemia.

## Data Availability

The datasets used and/or analyzed during the current study are available from the corresponding author upon reasonable request.
